# Myocardial iron quantification using T2-prepared SSFP parametric images at 3 Tesla

**DOI:** 10.1186/1532-429X-15-S1-P138

**Published:** 2013-01-30

**Authors:** Gabriel C Camargo, Tamara Rothstein, Flavia P Junqueira, Peter Kellman, Andreas Greiser, Ralph Strecker, Elsa Fernandes, Joao A Lima, Ronaldo SL Lima, Ilan Gottlieb

**Affiliations:** 1 CDPI - Clínica de Diagnóstico por Imagem, Rio de Janeiro, Brazil; 2 Laboratory of Cardiac Energetics, National Institutes of Health, Bethesda, MD, USA; 3 Siemens LTDA, São Paulo, Brazil; 4 Siemens Healthcare, Erlangen, Germany; 5 Medicine/Cardiology, Johns Hopkins University, Baltimore, MD, USA

## Background

Quantification of myocardial iron overload is critical for the management of patients with hemochromatosis. The effects of excess iron over T2 and T2* relaxation times are well known and both measures strongly correlate with iron concentration. Due to its lower sensitivity to B0 inhomogeneities, T2 has theoretical advantages over T2*, but the latter became the clinical standard as it can be easily obtained in a fast one breath-hold ECG gated multi-echo GRE sequence. T2* is especially challenging at 3T due to greater B0 inhomogeneities at higher field strengths. We aimed to validate a recently developed T2-prepared SSFP sequence that quantifies myocardial T2 times at 3T, compared to standard GRE based multi-echo T2* times at 1.5T.

## Methods

**Figure 1:**
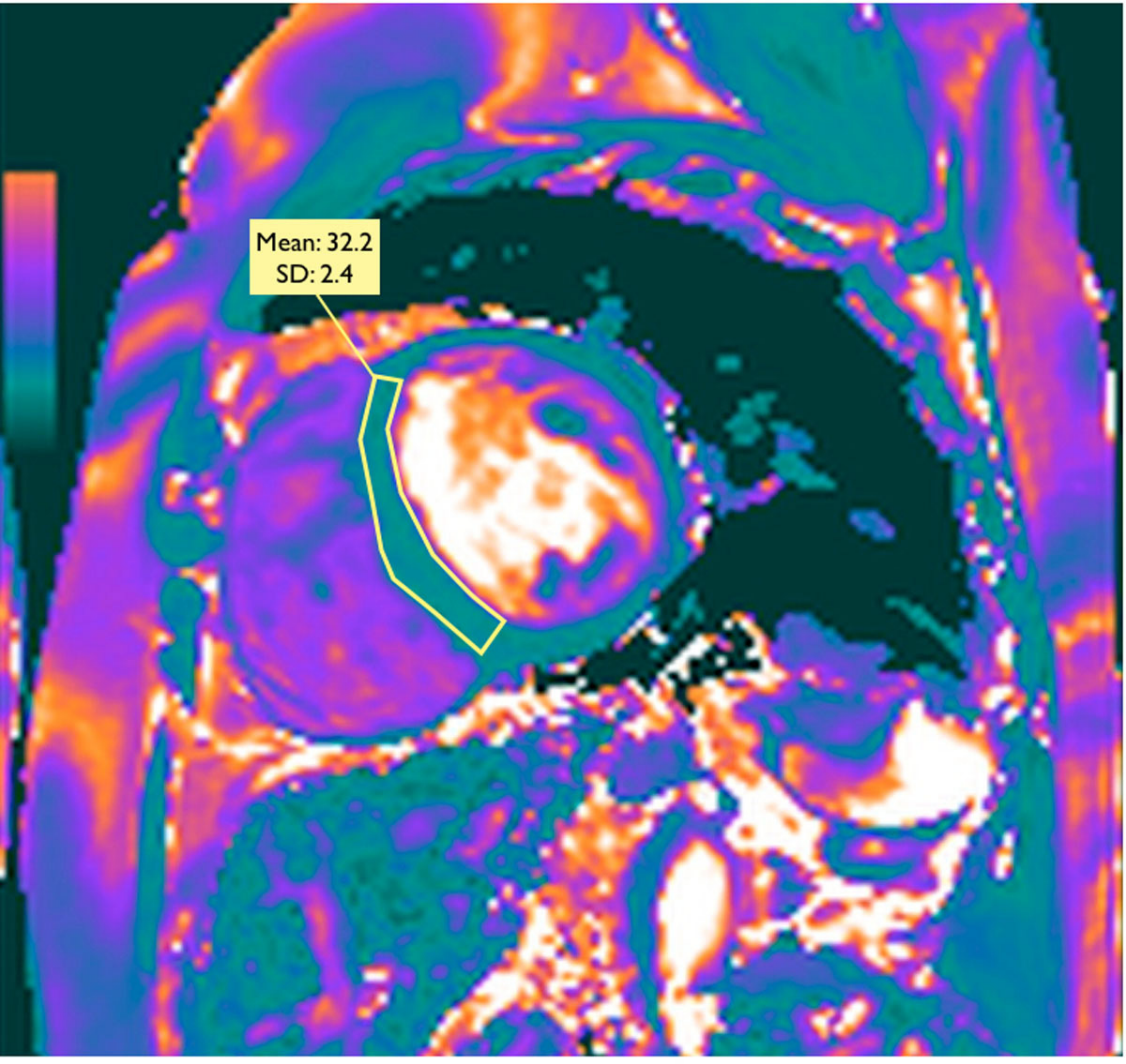
T2 map at 3T of a patient with iron overload showing reduced T2 time within the interventricular septum (32.2 ms), in agreement with a significantly reduced T2* time at 1.5T (8.5 ms - not shown).

A total of 15 normal volunteers and 7 chronic anemia patients (with a myocardial T2* measure <20 ms in the last 2 years, five of these on iron chelating therapy) were prospectively enrolled. Myocardial T2* and T2 times were quantified in the same day, the former using a breath-hold multi-echo GRE sequence at 1.5T (Symphony, Siemens, Erlangen, Germany) and the latter using a recently developed T2 mapping technique based on a breath-hold T2-prepared SSFP sequence at 3T (Verio, Siemens, Erlangen, Germany). All ROIs were placed at mid-interventricular septum, carefully avoiding the blood pool (Figure 1). All analyses were blinded.

## Results

**Figure 2:**
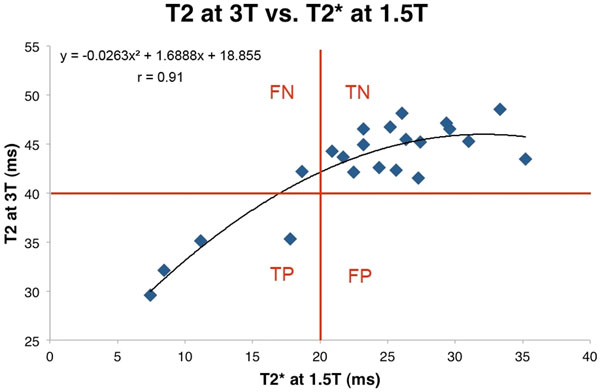
Correlation curve between T2 at 3T and T2* at 1.5T. The whole data were best fitted by a quadratic curve with r=0.91. Red lines delimitate true positives (TP), true negatives (TN), false positives (FP) and false negatives (FN) based on a T2 cut-off of 40 ms for the prediction of a T2* < 20 ms.

All patients had regular heart rhythm and all MRI exams showed diagnostic image quality. Volunteers and patients had significantly different mean myocardial T2* (27.2 ms +/- 3.9 vs. 15.4 ms +/- 6.3 p<0.05 respectively) and T2 times (44.9 ms +/- 2.2 vs. 37.9 ms +/- 6.6 p<0.05 respectively). 3T T2 times strongly correlated with 1.5T T2* times (r=0.91 and Figure 2). C-statistic of 3T T2 times for the prediction of a 1.5T T2* <20 ms was 0.97. Using the 3T T2 cut-off of 40 ms and the standard 1.5T T2* of 20 ms, sensitivity and specificity for 3T T2 were 80% and 100% respectively.

## Conclusions

Our results show that myocardial T2 values obtained with a T2-prepared SSFP parametric sequence can potentially serve as a valuable tool for quantification of iron overload at 3T.

## Funding

Internal.

